# Proteomic and metabolomic profiles of larval hemolymph associated with diapause in the cotton bollworm, *Helicoverpa armigera*

**DOI:** 10.1186/1471-2164-14-751

**Published:** 2013-11-01

**Authors:** Qi Zhang, Yu-Xuan Lu, Wei-Hua Xu

**Affiliations:** State Key Laboratory of Biocontrol, School of Life Sciences, Sun Yat-Sen University, Guangzhou, China

## Abstract

**Background:**

Diapause is programmed developmental arrest coupled with the depression of metabolic activity and the enhancement of stress resistance. Pupal diapause is induced by environmental signals and is prepared during the prediapause phase. In the cotton bollworm, *Helicoverpa armigera*, the prediapause phase, which contains two sub-phases, diapause induction and preparation, occurs in the larval stage. Here, we performed parallel proteomic and metabolomic analyses on *H. armigera* larval hemolymph during the prediapause phase.

**Results:**

By two-dimensional electrophoresis, 37 proteins were shown to be differentially expressed in diapause-destined larvae. Of these proteins, 28 were successfully identified by MALDI-TOF/TOF mass spectrometry. Moreover, a total of 22 altered metabolites were found in diapause-destined larval hemolymph by GC-MS analysis, and the levels of 17 metabolites were elevated and 5 were decreased.

**Conclusions:**

The proteins and metabolites with significantly altered levels play different roles in diapause-destined larvae, including diapause induction, metabolic storage, immune response, stress tolerance, and others. Because hemolymph circulates through the whole body of an insect, these differences found in diapause-destined larvae most likely correspond to upstream endocrine signals and would further influence other organ/tissue activities to determine the insect’s fact: diapause or development.

**Electronic supplementary material:**

The online version of this article (doi:10.1186/1471-2164-14-751) contains supplementary material, which is available to authorized users.

## Background

The process of developmental arrest in insects, known as diapause, is a critical mechanism that allows for individual survival in harsh environmental conditions. Like hibernating mammals, diapausing insects can resist environmental stress by depressing metabolic activity and enhancing resistance to adversity. Diapause has evolved as a basic strategy for survival. Temperature, photoperiod, and nutrition are common environmental signals that induce diapause [1]. Diapause is usually divided into three successive phases: prediapause, diapause, and postdiapause. The pre-diapause phase is further divided into two sub-phases: the induction phase and the preparation phase [2]. Environmental signals are perceived by insects in the prediapause phase, and physical signals are than transduced into humoral factors that trigger the decision to enter diapause. Individuals destined for diapause undergo subsequent physiological and metabolic changes, including the storage of additional energy reserves, the deposition of extra layers for waterproofing the cuticle, and the synthesis of storage proteins in the hemolymph. Changes that take place during the prediapause phase are vital to individual survival. However, little is known about the molecular mechanisms behind these physiological and metabolic shifts in the prediapause phase [3].

The cotton bollworm, *Helicoverpa armigera* (Noctuiadae, Lepidoptera), an agriculturally important pest, enters diapause at the pupal stage. Diapause is induced in this species by low temperature and short daylength during larval development. The prediapause phase in *H. armigera* occurs during the fifth and sixth instars: the induction phase bridges the fifth instar to the early sixth instar, and the preparation phase takes place during the mid-to-late stage of the sixth instar [4]. We have previously performed proteomic and metabolomic analyses of nondiapause- and diapause-destined larval brains from *H. armigera*. We found that proteins and metabolites with significantly altered levels in diapause-destined individuals during the induction phase are most likely involved in a special memory mechanism used for the storage of photoperiodic information, and those during the preparation phase are most likely involved in the regulation of specific accumulation for energy reserves [5]. However, it is not yet known what occurs in the hemolymph as a result of these memory signals. In the present study, we focus on the changes in the proteomic and metabolomic profiles of the hemolymph in diapause-destined larvae of *H. armigera* in the prediapause phase. Hemolymph is present in the open circulatory system in insects to provide intermediary metabolites for individual growth and development, as all tissues or organs are exposed to hemolymph. The hemolymph also plays important roles in hormone and nutrient transport, energy deposition, innate immune responses, waste removal, and water balance.

There are no obvious phenotypic differences to be observed between nondiapause- and diapause-destined larvae because both types of larvae have the same phenotypes of growth, development, and metamorphosis. Thus, it is difficult to identify genes that are specifically expressed or metabolites that are uniquely produced in diapause-destined larval hemolymph. Combined large-scale studies, such as transcriptomics, proteomics, and metabolomics, are particularly useful tools for the study of interactions between organisms and their environments. Recently, both proteomic and metabolomic techniques have been used successfully in the study of insect diapause [6–10]. Currently, however, little is known about the proteomic and metabolomic differences between hemolymph in nondiapause- and diapause-destined larvae in the prediapause phase. To understand the molecular events that occur in the prediapause phase, larval hemolymph from *H. armigera* was investigated by parallel proteomic and metabolomic analyses. These experiments were conducted on hemolymph from four stages of the prediapause phase: the mid-late stage of the fifth instar, the early stage of the sixth instar (belonging to the diapause induction phase), and the middle and the late stages of the sixth instar (belonging to the diapause preparation phase). A total of 37 differentially expressed proteins and 22 altered metabolites were identified, and these data provided valuable insights into the molecular characteristics of diapause induction and preparation.

## Results

### Two-DE analysis of differentially expressed proteins

**Figure 1:**
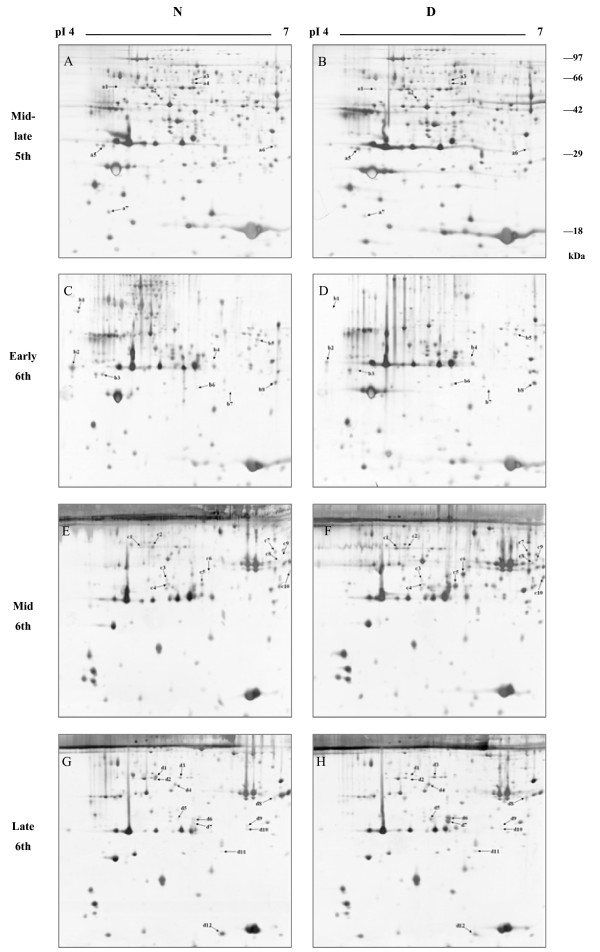
**Representative 2-DE images of the proteins from** ***H. armigera*** **larval hemolymph.** Proteins were extracted from nondiapause- (N) and diapause-destined (D) larval hemolymph at four stages: **(A and B)** mid-late stage of the fifth instar, **(C and D)** early stage of the sixth instar, **(E and F)** middle stage of the sixth instar, and (G and H) late stage of the sixth instar. Proteins were separated by IEF (18 cm IPG strips, pH 4–7, L) and 12% SDS-PAGE. Gels were stained by MS-compatible silver stain. Based on triplicate replications, only statistically significant (*p*-value < 0.05) protein spots that changed ≥1.5-fold were considered for further analysis. Differentially expressed protein spots are marked with letters and numbers. Letters 'a’, 'b’, 'c’, and 'd’ represent the mid-late stage of the fifth instar, the early, the middle, and the late stages of the sixth instar, respectively.

**Figure 2:**
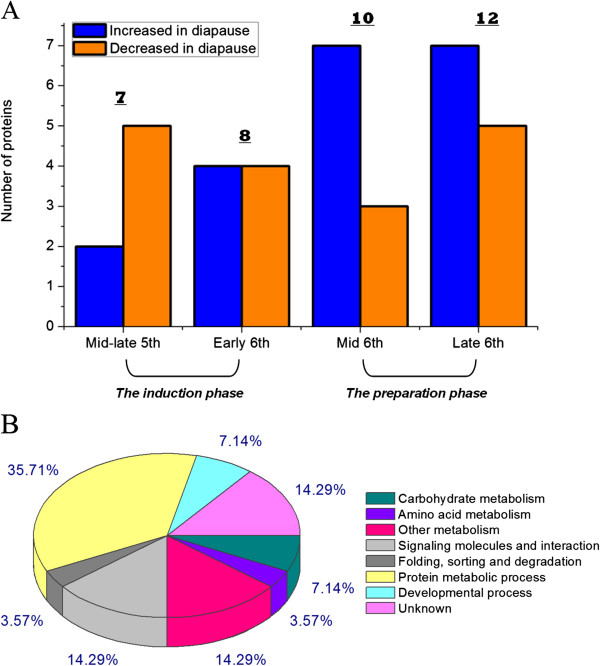
**Number of differentially expressed proteins (A) and classification of the identified proteins (B) in** ***H. armigera*** **larval hemolymph in the prediapause phase.**

Two-dimensional electrophoresis (18 cm IPG strips, pH 4–7, linear) was combined with silver staining for investigations of the proteomic differences in hemolymph at four larval stages: a) the mid-late stage of the fifth instar, b) the early stage of the sixth instar (diapause induction phase), c) the middle stage, and d) the late stage of the sixth instar (diapause preparation phase). Quantitative analysis revealed that 37 differentially expressed proteins were found in all three biological replicates in diapause-destined larval hemolymph during the prediapause phase; these proteins are marked with small arrows in the gel images (Figure 1). In the diapause induction phase, 2 protein spots were more prominent (spots no. a1, a5) and 5 were less intense (spots no. a2-a4, a6, a7) in the mid-late stage of the fifth instar. Four protein spots were more intense (spots no. b4, b6-b8) in the early sixth instar, while 4 were less intense (spots no. b1-b3, b5). In the diapause preparation phase, 7 protein spots were more pronounced (spots no. c1, c5-c10) and 3 were less prominent (spots no. c2-c4) in the middle stage of the sixth instar. Seven protein spots were more intense (spots no. d3-d8, d11) in the late stage of the sixth instar, and 5 were less intense (spots no. d1, d2, d9, d10, and d12). Thus, a total of 15 differentially expressed proteins were detected in the induction phase and 22 were detected in the preparation phase (Figure 2A).

### Protein identification and classification

The 37 differentially expressed proteins were excised from the gels and identified through MALDI-TOF/TOF mass spectrometry. Representative PMF and MS/MS spectra of protein spot b3 are shown in Additional file 1: Figure S1. The MS/MS data were searched against an in-house *H. armigera* EST database constructed in our laboratory, the NCBInr database or the NCBI EST_others database. A total of 17 proteins were successfully identified in the EST_Har database, and 11 proteins were identified in either the NCBInr database or the NCBI EST_others database (Table 1; Additional file 2: Figure S2). As shown in Table 1, several different spots in a gel were identified as the same protein. For example, both protein spots a3 and a4 were identified as glucosamine (N-acetyl)-6-sulfatase precursor, which is most likely a result of posttranslational modification. Using this method, a total of 28 differentially expressed proteins were identified in larval hemolymph. Because there are no commercial antibodies against these differentially expressed proteins from *H. armigera*, validation experiments such as western blot analysis were not performed in this study.

**Table 1:** **Differentially expressed proteins identified by MALDI-TOF/TOF**

Spot no.	Protein name	Organism	NCBI accession no.	Theoretical MW(kDa)/pl	Fold change	Database	Mowse score^a^	No. of peptides	EC no.
Proteins increased in diapause larval hemolymph									
a1	Serpin 3a	*Manduca sexta*	gi|27733415	51.13/5.26	2.8	EST_Har	311	4	-
a5	Ommochrome binding protein	*M. sexta*	gi|400673	30.61/5.89	1.5	EST_Har	369	4	-
b6	Glutathione S-transferase	*Helicoverpa amigera*	gi|297340786	23.72/6.18	2.8	EST_Har	202	3	EC:2.5.1.18
b7	Hydroxypyruvate isomerase	*Danaus plexippus*	gi|357604475	29.12/6.00	specific	EST_others	151	2	EC: 5.3.1.22
b8	Pyridoxine 5′-phosphate oxidase	*Bombyx mori*	gi|112984194	29.87/8.76	1.5	EST_Har	120	2	EC:1.4.3.5
c5	C-type lectin 7	*H. armigera*	gi|385202653	34.99/5.98	2.3	EST_Har	116	2	-
c7	Serpin	*H. armigera*	gi|314912147	43.53/5.04	2.7	EST_Har	92	2	-
c9	Imaginal disc growth factor-like protein	*Mamestra brassicae*	gi|85726208	47.83/7.08	3.8	NCBI	99	1^b^	EC:3.2.1.14
d4	Beta-1,3-glucan recognition protein 3	*H. armigera*	gi|208972535	53.37/5.85	1.9	NCBI	262	4	EC:3.2.1-
d5	Putative serine protease-like protein 2	*Lonomia obliqua*	gi|56462300	32.15/5.79	5.9	EST_others	182	2	EC:3.4.21.-
d6	C-type lectin 7	*H. amigera*	gi|385202653	34.99/5.98	7.4	EST_Har	128	2	-
d7	C-type lectin 7	*H. amigera*	gi|385202653	34.99/5.98	5.5	EST_Har	102	2	-
d8	Serpin	*H. amigera*	gi|314912147	43.53/5.04	2.9	EST_Har	165	2	-
d11	Ferritin	*M. sexta*	gi|7272336	26.51/5.44	2.6	EST_Har	193	3	-
**Proteins decreased in diapause-destined larval hemolymph**									
a2	Growth-blocking peptide binding protein	*Heliosthis virescens*	gi|238915973	23.63/5.16	1.7	EST_others	79	1	-
a3	Sulfatase	*D. plexippus*	gi|357631807	56.65/5.34	2.2	EST_others	105	1	EC:3.1.6.14
a4	Sulfatase	*D. plexippus*	gi|357631807	56.65/5.34	2.3	EST_others	114	1	EC:3.1.6.14
a6	EndoU protein, partial	*Popilio polytes*	gi|389615052	28.70/8.38	3.3	EST_Har	217	4	EC:3.1--
a7	Kazal-type inhibotor	*D. plexippus*	gi|357625979	22.97/5.07	1.8	EST-Har	229	2	-
b1	Protein disulfide isomerase	*B. mori*	gi|112984454	55.59/4.60	5.7	EST_others	140	2	EC:5.3.4.1
b3	Ommochrome-binding protein	*M. sexta*	gi|400673	30.61/5.89	2.8	EST_Har	207	3	-
b5	Hemolymph proteinase 18	*M. sexta*	gi|56418417	44.14/5.94	2.0	EST_Har	49	1	EC:3.4.21-
c2	Serine proteinase-like protein 1	*H.armigera*	gi|208972549	46.51/5.43	1.8	NCBI	129	3	EC:3.4.21-
c3	C-type lectin	*H. armigera*	gi|106897087	38.01/5.42	1.9	EST_Har	49	1	-
c4	C-type lectin	*H. armigera*	gi|106897087	38.01/5.42	2.9	EST_Har	118	1	-
d1	Serine proteinase-like protein 1	*H. armigera*	gi|208972549	46.51/5.43	10.7	NCBI	84	2	EC:3.4.21-
d2	Serine proteinase-like protein 1	*H. armigera*	gi|208972549	46.51/5.43	10.7	NCBI	148	3	EC:3.4.21-
d12	Unknown secreted protein	*Papilio xuthus*	gi|389609461	17.50/8.55	2.3	EST_Har	195	3	EC:3.4.21

^a^Mowse scores greater than the significance threshold indicate identity or extensive homology (p < 0.05). Three database have their respective significance thresholds, the EST_Har database is 31, the NCBInr database is 46, and the NCBI EST_others database is 57.^b^Additional data for single peptide-based identifications are presented in Additional file 2: Figure S2.

The 28 identified proteins were classified according to the KEGG pathway maps or subordinately according to their biological processes from Gene Ontology. Proteins that were assigned functions, including glycan biosynthesis and metabolism, metabolism of cofactors and vitamins according to the KEGG pathway maps, or those related to metabolic processes according to Gene Ontology, were merged into a class referred to as “other metabolism”. Among the differentially expressed proteins, 36% (10/28) were categorized as being involved in protein metabolic process. An additional 25% (7/28) were classified as being involved in metabolism of nutrients including carbohydrate metabolism, amino acid metabolism and other metabolism. Another 14% (4/28) were categorized as signaling molecules and interaction (Figure 2B).

### GC-MS analysis of significantly changed metabolites

**Figure 3:**
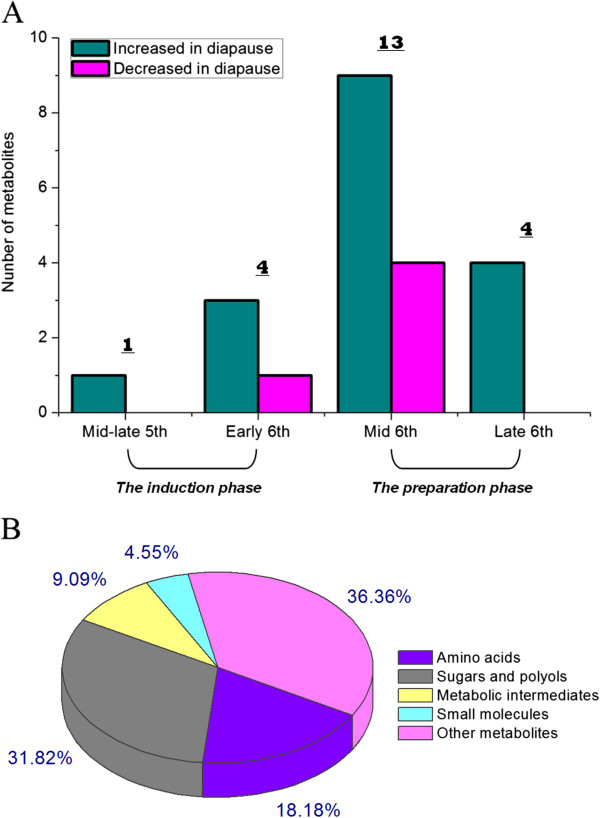
**Number (A) and classification (B) of significantly changed metabolites in** ***H. armigera*** **larval hemolymph in the prediapause phase.**

Comparative metabolomic analysis was also carried out between hemolymph samples from nondiapause- and diapause-destined *H. armigera* larvae in the prediapause phase. Metabolites were extracted from larval hemolymph at four stages, as described above, and analyzed by GC-MS. Representative GC-MS total ion chromatograms depicting the metabolite profiles of larval hemolymph are shown in Additional file 3: Figure S3. A total of 59 metabolites were identified across all four stages, including 18 amino acids, 12 sugars and polyols, 6 metabolic intermediates, 3 small molecules, and 20 other metabolites (Table 2). Based on four biological replicates, 22 metabolites exhibited significant changes in abundance in diapause-destined larvae. In the mid-late stage of the fifth instar, the only significant difference was found for phosphorylethanolamine, as its content was increased in diapause-destined larvae. In the early stage of the sixth instar, 4 metabolites were detected at altered concentrations. Levels of trehalose, glucose, and mevalonic acid were higher, while less histidine was present. Thirteen metabolites were present at changed levels in the middle stage of the sixth instar: 9 were found at higher concentrations, and 4 were detected at lower concentrations. γ-Aminobutyric acid (GABA) was only detected in diapause-destined larvae, and other 8 metabolites were present at higher concentrations in diapause-destined larvae: glycine, fructose, glucose, glycerol, ribitol, pyruvate, urea, and phosphoric acid. The concentrations of ornithine, 3-hydroxy-3-methylglutaric acid, phosphorylethanolamine, and ribonic acid were decreased. In the late stage of the sixth instar, all 4 metabolites with altered profiles in diapause-destined larvae were found at higher concentrations: *N*-Acetylglucosamine, glucose, *N*-Acetylglutamate, and gluconic acid. As shown in Figure 3A, more metabolites showed altered concentrations in the diapause preparation phase than in the diapause induction phase; this result is consistent with the proteomic analysis above. Among the metabolites with altered profiles (Figure 3B), other metabolites accounted for 36% (8/22) of the total. Sugars and polyols represented 32% (7/22) of the total, and amino acids made up another 18% (4/22).

**Table 2:** **Metabolites identified in tha larval hemolymph from** ***H.armigera***

Metabolite	Fold change of relative ratio^a^			
	Mid-late 5th	Early 6th	Mid 6th	Late 6th
**Amino acid**				
Alanine	ns^b^	ns	ns	ns
β-Alanine	ns	ns	ns	ns
Glutamate	ns	ns	ns	ns
Glycine	ns	ns	**1.7**	ns
Histidine	ns	**-4.5**	-^c^	-
Homoserine	ns	ns	-	-
Isoleucine	ns	ns	ns	ns
Leucine	ns	ns	ns	ns
Methionine	ns	ns	ns	ns
*N-*Acetylglutamate	ns	ns	-	**1.9**
Ornithine	ns	ns	**-2.4**	ns
Phenylalanine	ns	ns	ns	ns
Proline	ns	ns	ns	ns
Serine	ns	ns	ns	ns
Threonine	ns	ns	ns	ns
Tryptophan	-	-	-	ns
Tyrosine	ns	ns	ns	ns
Valine	ns	ns	ns	ns
**Sugar and polyols**				
Arabinitol	ns	ns	-	-
Cholesterol	ns	ns	ns	ns
Erythritol	ns	ns	-	-
Fructose	ns	ns	**3.8**	ns
Galactitol	ns	ns	-	-
Glucose	ns	**1.7**	**3.0**	**3.1**
Glycerol	ns	ns	**2.2**	ns
Myo-inositol	ns	ns	ns	ns
Ribitol	ns	ns	**2.4**	-
Sitosterol	ns	ns	ns	ns
Trehalose	ns	**8.3**	ns	ns
Turanose	-	ns	ns	ns
**Metabolic intermediates**				
Fumaric acid	ns	ns	ns	ns
Isocitric acid	ns	ns	ns	ns
Malic acid	ns	ns	ns	ns
Pyruvate	ns	ns	**1.5**	ns
Succinic acid	ns	ns	ns	ns
Urea	ns	ns	**2.5**	-
**Small molecules**				
Free amine	ns	ns	ns	ns
Free phosphate	ns	ns	ns	ns
Phosphoric acid	ns	ns	**1.5**	ns
**Other metabolites**				
2-Hydroxymandelic acid	-	-	ns	ns
3-Aminoisobutyric acid	ns	ns	ns	ns
3-Hydroxy-3-methylglutaric acid	-	-	**-2.7**	ns
3-Hydroxypropanoic acid	ns	ns	-	-
	ns	ns	ns	ns
Galatonic acid	ns	ns	-	-
γ-Aminobutyric acid	ns	ns	**D.specific** ^**d**^	ns
Gluconic acid	ns	ns	ns	**1.7**
Glyceric acid	-	ns	-	-
Linoleic acid	-	ns	-	-
Methylcitric acid	-	ns	ns	ns
Mevalonic acid	ns	**1.7**	-	ns
*N*-Acetylglucosamine	ns	ns	-	**5.3**
Octadecenoic	-	-	ns	ns
Palmitic acid	ns	ns	ns	ns
Pantothenic acid	ns	-	ns	ns
Phosphorylethanolamine	**1.5**	ns	**-2.4**	ns
Ribonic acid	ns	ns	**-2.9**	ns
Stearic acid	-	-	ns	ns
Threonic acid	ns	ns	-	-

^a^ Fold change with a positive number indicates metabolic levels were significantly increased in diapause-destine larval hemolymph, while a negative number indicates metabolite levels were significantly decreased in destined diapause-destine larval hemolymph. Based on four biological replicate analyses, only metabolites that changes ≥1.5-fold in relative ratios (p-value < 0.05) were considered for further analysis, and were marked in bold. ^b^ ns, no significant difference. ^c^-, metabolite was found in less than three measurements and wasn’t able to be quantified accurately. ^d^ Metabolite was only found in dispause-destined larval hemolymph.

**Figure 4:**
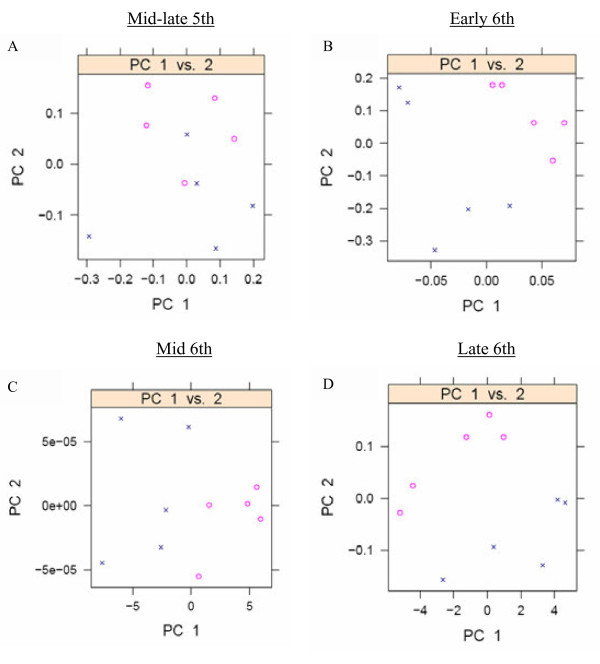
**Principal component analysis score plots of the hemolymph metabolites by comparison of nondiapause- (circle) and diapause-destined (cross) larvae in the prediapause phase at four stages: (A) mid-late stage of the fifth instar, (B) early stage of the sixth instar, (C) middle stage of the sixth instar, and (D) late stage of the sixth instar.**

The results of principal component analysis (PCA) carried out on the 59 identified metabolites suggest that there are no obvious physiological differences between the nondiapause- and diapause-destined fifth instar larvae (Figure 4A). However, physiological differences are observed in the sixth instar larvae (Figure 4B, 4C, and 4D) by PC1 or PC2.

## Discussion

Prediapause phase is a key period during which insects determine whether to develop into adults without interruption or to enter diapause at the pupal stage. Taking the latter path requires changes in the individual’s metabolic pathway to elevate energy reserves and produce cryoprotectants to defend against low temperature. However, little is known about the relationship between environmental signals and the expression of the diapause phenotype [3]. A major reason for this lack of knowledge is the fact that no obvious phenotypic changes can be observed in diapause-destined individuals during the prediapause phase. In this study, we combined proteomic and metabolomic approaches to investigate changes in the expression of proteins and metabolites between larval hemolymph from nondiapause- and diapauses-destined *H. armigera*. Significantly changed proteins and metabolites were identified and analyzed, and our results revealed important clues regarding the diapause induction and preparation in the prediapause phase. It is well known that the short daylength is the major factor behind the effective induction of diapause, while low temperature is an associated minor factor. We believe that the altered levels of proteins and metabolites in hemolymph are the result of a cooperative influence of photoperiod and temperature because we used short daylength and low temperature in our experiment.

### Diapause induction

Pyridoxine 5′-phosphate oxidase, an enzyme that is increased in the early stage of the sixth instar, catalyzes the biosynthesis of pyridoxal 5′-phosphate [11]. The vitamin B6 derivative pyridoxal 5′-phosphate acts as a coenzyme and participates in the biosynthesis of some neurotransmitters, such as serotonin, dopamine, and GABA [12]. Biogenic amine neurotransmitters in hemolymph, including serotonin and dopamine, have been shown to be involved in insect diapause in *Mamestra brassicae* and in *Pieris brassicae*[13, 14]. In the middle stage of the sixth instar, GABA was only detected in diapause-destined larval hemolymph. GABA is a major inhibitory neurotransmitter in insects. It has been reported that GABA and the GABA receptor antagonist picrotoxin influenced the incidence of diapause in *Sarcophaga bullata*[15]. GABA was also found to repress diapause hormone (DH) secretion, leading to diapause in *Bombyx mori*[16]. We speculate that the high concentration of GABA in diapauses-destined *H. armigera* larvae may be involved in inhibiting neurohormone secretion. This leads, in turn, to changes in larval metabolism and finally causes insects to enter diapause. Therefore, the increased pyridoxine 5′-phosphate oxidase in the diapause induction phase is most likely a response to the short daylength signal to regulate endogenous chemicals such as neurotransmitters for insect diapause.

The amino acid histidine is a precursor to histamine, and the histidine levels were dramatically decreased in the early stage of the sixth instar. There is much evidence to suggest that histamine acts as a neurotransmitter released from the photoreceptor synapse of insects and other arthropods. Histamine acts by directly gating chloride channels and inhibits the activity of central nervous circuits in arthropods [17, 18]. Diapause is mostly induced in *H. armigera* by short daylength signals which are received by photoreceptors in the brain. Significant changes in the levels of histidine most likely indicate altered levels of the photoreceptor transmitter histamine, which is likely to participate in the molecular events of the diapause induction phase.

### Metabolic storage

Diapause-destined individuals must store additional metabolic reserves such as fuel reserves and cryoprotectant molecules because adequate reserves are critical to allowing individuals to sustain a basic metabolism in the harsh environmental conditions that occur during diapause. The major energy resource glucose was maintained at a constant high level during the sixth instar in diapause-destined larvae. Fructose and pyruvate levels were also elevated in the middle stage of the sixth instar. In our previous study, glucose and pyruvate levels decreased in diapausing pupae whose metabolic activities were dramatically reduced [9]. Accordingly, accumulation of these glycolysis-related metabolites in diapause-destined larvae implies increased energy reserves *in vivo*. Interestingly, trehalose, as an important cryoprotectant and energy resource that usually accumulates at the pupal stage [19], was heavily elevated in the early stage of the sixth instar.

On the other hand, there is significant evidence to suggest that synthesis of cryoprotectants is associated with insect diapause [20]. Glycerol and ribitol are important cryoprotectant molecules found in insects [21]. These two cryoprotectants were found to accumulate in the diapausing flesh fly, *Sarcophaga crassipalpis*[22], and in the rice stem borer, *Chilo suppressalis*[23]. In this study, both compounds were found to be elevated in the middle stage of the sixth instar, showing that polyol concentrations in diapause individuals are closely correlated with cold resistance. Two major sugars, glucose and fructose, were also elevated in the sixth instar. These two sugars may also be used as precursors for the synthesis of cryoprotectants [24].

Urea levels were increased in diapause-destined larval hemolymph in the middle stage of the sixth instar. On the other hand, the levels of ornithine, which serves as precursor of urea, were decreased. The concentrations of *N*-Acetylglutamate, an activator of carbamoyl phosphate synthetase in the urea cycle [25], were also increased in diapause-destined larvae during the diapause preparation phase (the late sixth instar). It has been reported that the overwintering wood frog, *Rana sylvatica*, can accumulate additional urea, suggesting that the organic osmolyte also functions as a cryoprotectant such as glycerol in terrestrially hibernating amphibians [26]. Thus, urea in diapause-type insects may contribute to cold resistance.

Although both nondiapause- and diapause-destined larvae were reared at the same temperature (20°C) with different photoperiods, diapause-destined individuals could accumulate additional polyols and sugars. This result indicates that the synthesis of cryoprotectants and energy reserves is mainly attributable to short daylength conditions in this study, and the photoperiod is a key factor influencing the induction of diapause. Experiments in other insect species also showed this special linkage between photoperiod and cold-hardiness [20].

### Immune response

Insects are vulnerable to fungal and bacterial infections during diapause. The innate immune system continues to function in diapausing individuals and plays an important role in preventing the invasion of the body by microorganisms [27]. Serine proteases participate in many aspects of invertebrate immunity, including hemolymph coagulation, antimicrobial peptide synthesis, and melanin synthesis [28]. The activity of these enzymes is regulated by serine protease inhibitors (serpins). Thus, serpins in insect hemolymph also function to protect individuals from infection [29]. The expression of serine protease-like protein 2, serpin 3a, and serpin was found to be increased in diapause-destined individuals. On the other hand, levels of Kazal-type inhibitor, hemolymph proteinase 18 (a serine proteinase identified in *Manduca sexta* hemolymph), and serine proteinase-like protein 1 were decreased in these individuals. Serine protease-like protein 2 and serpin, which accumulate at the late sixth instar of diapause-destined larvae, may be used in the long diapause phase to prevent invasion by microorganisms. This function has been reported for *Nasonia vitripennis*[30], *Sesamia nonagrioides*[7], *Leptinotarsa decemlineata*[31], *Delia antiqua*[32], and *Culex pipiens*[33], indicating that serine protease-like protein 2 and serpin are most likely closely linked to insect diapause. We speculate that the serine proteinase-like protein 1 may not be suitable to act in diapausing individuals at low temperature, and the expression of this protein is consequently decreased in the late sixth instar of diapause-destined larvae.

Two different C-type lectins exhibited significantly altered expression in the diapause preparation phase. The expression of C-type lectin 7 was increased at the late stage of the sixth instar in diapause-destined larvae, while the expression of another C-type lectin was decreased. C-type lectins are calcium-dependent carbohydrate-binding pattern recognition proteins. Lepidopteran C-type lectins have been shown to function in innate immune responses such as phagocytosis, prophenoloxidase activation, and hemocyte nodule formation [34, 35]. C-type lectin 7, which accumulates at the late stage of the sixth instar in diapause-destined larvae, may be highly stable at low temperature and can be used by diapausing individuals. Pattern recognition proteins can recognize foreign invading microorganisms, which is the crucial first step in producing an immune response [36]. A pattern recognition protein, beta-1,3-glucan recognition protein 3 (βGRP3), was found to exhibit increased expression at the late stage of the sixth instar in diapause-destined larvae. Fungal infection can be recognized by βGRP through its binding to beta-1,3-glucan on the surface of fungal cell walls. Subsequently, βGRP may work in concert with serine proteases to trigger the prophenoloxidase activation pathway, which is an important defense against microbial invasion in insects [37].

Previous studies on serine proteases, protease inhibitors and lectins in hemolymph have revealed their functions in immune response for morphogenesis. Because both diapause- and nondiapause-type individuals must undergo a molting process, high expression levels of proteases, protease inhibitors, and lectins in these two types of individuals are correlated with morphogenesis. However, high expression levels of these proteins only in diapause-type individuals may serve in immune responses to facilitate survival in adverse environmental conditions over the long diapause period.

### Stress tolerance

One of the most common features in insect diapause is enhanced stress tolerance, which includes resistance to highly reactive chemical species, desiccation, ultraviolet radiation, salt, and other environmental or physiological stresses [38]. The expression of several stress tolerance-related proteins was significantly altered in the larval hemolymph of diapause-destined individuals. The expression of glutathione S-transferase (GST) was increased in the early stage of the sixth instar larvae. We showed that both GST mRNA and protein were heavily expressed in pupal brains of *H. armigera* at diapause initiation [6, 39]. It has been reported that diapause hormone expressed at the larval stage of *B. mori*, as an intensified memory, can subsequently induce high expression of diapause hormone at the pupal stage [40]. Therefore, we speculate that GST expressed in the early stage of the sixth instar larvae may cause high GST expression at the pupal stage. GST has antioxidant activity and can protect cellular components of diapause individuals from oxidative damage, and thus, the accumulation of GST in diapause individuals is most likely involved in an oxidative stress response [41]. The levels of ferritin were also increased in the late stage of the sixth instar. High abundance of ferritin was also found in other diapause-destined insects such as *N. vitripennis*[30] and *Calanus finmarchicus*[42], and in the pupal brains of *H. armigera* at diapause initiation [39]. It has been shown that the concentration of ferritin increases in response to stresses such as anoxia [43]. Ferritin serves as an important ion storage protein in hemolymph and functions in maintaining the iron balance. The accumulation of ferritin can prevent destructive oxidative reactions by mitigating iron toxicity [44].

In addition, *N*-Acetylglucosamine (GlcNAc) levels were found to be elevated in diapause-destined larvae in the late stage of the sixth instar. GlcNAc is the monomeric unit of the polymer chitin, which is one of the major components of insect cuticle [45]. Cuticle structural components are enriched in the diapause preparation phase to enhance waterproofing and to promote survival [46]. Furthermore, increased pupal cuticle protein was observed during early adult diapause in the mosquito *C. pipiens*[47]. Elevated levels of GlcNAc most likely contribute to a harder cuticle in diapause individuals, improving tolerance to winter stresses and defenses against microbial invasion.

### Others

Mevalonic acid was increased in the early stage of the sixth instar. Mevalonic acid is a precursor to the mevalonate pathway, which is the early step of juvenile hormone (JH) synthesis in insects [48]. JH participates in the regulation of insect development, metamorphosis, diapause, and reproduction [1, 49, 50]. To date, no convincing experimental evidence exists to support the notion that JH plays a role in the induction of pupal diapause. Although major pulses of JH activity with a rhythmicity of 24 h were unique to pupal diapause-destined individuals in the flesh fly *S. crassipalpis*[51], the function of JH in the onset of pupal diapause requires further research.

An imaginal disc growth factor-like protein showed significantly increased expression in the middle stage of the sixth instar. Imaginal disc growth factors (IDGFs) are soluble growth factors that promote cell proliferation in imaginal discs. Several IDGFs have been identified from the fat body of *Drosophila*; these growth factors are secreted and transported to target tissues through hemolymph [52]. Thus, the IDGFs that accumulate in diapause-destined larval hemolymph may be used for the development of imaginal discs when diapause is broken.

A number of other proteins and metabolites showed significantly altered expression in diapause-destined larvae. These include sulfatase, endoU protein, protein disulfide isomerase, glycine, phosphorylethanolamine, and ribonic acid. Further research is required to reveal the roles of these proteins and metabolites in the regulation of diapause.

## Conclusions

In summary, we identified significantly changed proteins and metabolites in larval hemolymph of diapause-destined *H. armigera* using parallel proteomic and metabolomic analyses. In the prediapause phase, external environmental signals are received and processed by larval brain, and subsequently produced endocrine signals induce changes of proteins and metabolites in hemolymph. Proteins and metabolites that altered in the prediapause phase were involved in various aspects of diapauses: diapause induction, metabolic storage, immune response, stress tolerance, and others. These results here provided many valuable insights into the molecular characteristics of diapause induction and preparation. However, more research needs to be performed on the functions of altered proteins and metabolites in the future to understand the molecular mechanism in the prediapause phase.

## Methods

### Insects rearing and tissue collection

*H. armigera* was kindly provided by Dr. J.-Y. Su (Nanjing Agriculture University, China). Larvae were reared on an artificial diet at 20°C and long daylength conditions (14 L : 10 D) to generate non-diapause pupae, and at 20°C and short daylength conditions (10 L : 14 D) to generate diapause pupae (Additional file 4: Figure S4). Diapause was determined by the retention of eyespots in the postgenal region of pupa [53]. Larval hemolymph was collected into microcentrifuge tubes containing phenylthiourea by clipping off a proleg with microscissors. Each sample contained hemolymph pooled from 30 larvae. After centrifuged for 5 min at 12,000 *g* and 4°C, hemolymph samples were stored at -80°C until use.

### Protein extraction

Three biological replicates were prepared for proteomic analyses. Protein extraction was first performed by precipitating the hemolymph samples in 10% TCA containing 20 mM dithiothreitol (DTT) on ice for 1 h. After centrifuging at 12,000 *g* for 15 min, precipitate was washed three times with cold acetone (containing 20 mM DTT). The pellet was dried and resolved in 350 *μ*L rehydration buffer containing 7 M urea, 2 M thiourea, 4% (w/v) CHAPS, 40 mM DTT, 0.5% (v/v) IPG buffer (pH 4–7, linear), and 0.001% (w/v) bromophenol blue. Protein concentrations of the samples were determined by Bradford method [54].

### Two-dimensional gel electrophoresis (2-DE)

The hemolymph protein samples (340 *μ*L, containing approximately 1000 *μg*) were loaded onto 18 cm immobiline pH gradients (IPG) strips (pH 4–7, linear; GE Healthcare), and applied by in-gel rehydration overnight in the reswelling tray. The first dimension isoelectric focusing (IEF) was carried out in an IPGphor II (GE Healthcare) as follows: 200 V for 1 h, 500 V for 1 h, 1000 V for 2 h, gradient from 1000 V to 8000 V within 1 h, and 8000 V for 5 h. After IEF, the IPG strips were incubated for 15 min in equilibration buffer containing 6 M urea, 30% (w/v) glycerol, 2% (w/v) SDS, 50 mM Tris–HCl (pH 8.8), and 1% (w/v) DTT, and were incubated for another 15 min in the same equilibration buffer except that DTT was replaced with 2.5% (w/v) iodoacetamide. The equilibrated IPG strips were transferred to 12% SDS-polyacrylamide gels and sealed with agarose. The second dimensional electrophoresis was performed at 80 V for 4 h, 100 V for 4 h, and 120 V for 2 h. The gels were stained with a mass spectrometry compatible silver staining method, without glutaraldehyde in the sensitization solution. Three independent biological replicates experiments were performed.

### 2-DE image analysis

The stained gels were scanned with an Image Scanner III (GE Healthcare). The gel images were analyzed with ImageMaster 2D Platinum 7.0 according to the user manual provided by GE Healthcare. Spots were detected and matched automatically; manual edition was carried out when necessary. For comparison of spot quantities, spot volumes were normalized as a relative volume (% vol, a percentage of the total volumes of all of the spots in the gel). Based on triplicate analyses, spots that showed a statistically significant (*p*-value < 0.05, One-way ANOVA) and changed ≥1.5-fold were considered for further analysis.

### Mass spectrometry identification and database searching

Spots were excised manually and subjected to in-gel digestion. Before digestion, spots were washed twice in Milli-Q water and dehydrated with acetonitrile, then reswelled on ice for about 45 min in 5 *μ*L 25 mM NH_4_HCO_3_ digestion buffer containing 12.5 ng/μL porcine trypsin (Promega). Excess buffer that couldn’t absorb by the gel spots was removed and replaced with digestion buffer without trypsin. Digestion was carried out at 37°C overnight.

A sample (5 *μ*L) of each digest was applied directly to an AnchorChip plate (Bruker Daltonics), followed by 1 *μ*L matrix solution of 0.4 mg/mL α-cyano-4-hydroxy- cinnamic acid (HCCA) in 70% acetonitrile, and then 1 *μ*L TFA to remove remaining salt ions. Mass spectra were recorded on a Bruker Ultraflex III MALDI TOF/TOF mass spectrometer (Bruker Daltonics). Acquired MS and MS/MS spectra were sent as combined peak lists by BioTools software 3.1 (Bruker Daltonics) to Mascot search engine (Matrix Science), and searched against an in-house *H. armigera* EST database constructed in our laboratory (accession Nos. LIBEST_027843 NBR, LIBEST_027842 DBR, LIBEST_027820 NFB and LIBEST_027821 DFB) [9], the NCBInr database or the EST_others database. Only matches with significant scores (*p* < 0.05) were considered.

The identified proteins were submitted to Universal Protein Knowledgebase (UniProt) to obtain KEGG annotations, and then they were classified according to Kyoto Encyclopedia of Genes and Genomes (KEGG) pathway maps. Some of them could not be assigned in KEGG pathway maps, therefore they were classified according to the biological process of Gene Ontology.

### Metabolites extraction and derivatization

Four biological replicates of samples were prepared. For each sample (contained 100 *μ*L hemolymph), 800 *μ*L methanol and 100 *μ*L Milli-Q water (containing 0.1 mg sucrose as an internal control) were added. The mixture was mixed thoroughly on a vortex and incubated on ice for 10 min. The mixture was vigorously extracted once again for 2 min and incubated on ice for 2 h. After centrifugation at 20,000 *g* for 10 min, 600 *μ*L supernatant was collected to dry in a vacuum concentrator. The dried sample was dissolved in 90 *μ*L methoxylamine hydrochloride (15 mg/ml in pyridine), incubated for 16 h at room temperature, and trimethylsilylated in 90 *μ*L MSTFA/TCMS reagent (containing 1% TMCS) for 1 h. At last, 120 *μ*L hexane was added to stop the derivatization reaction.

### GC-MS analysis

One microliter of the derivatized sample was auto-injected at 1:10 split ratio into a Trace GC Ultra-DSQII GC-MS (Thermo) equipped with a DB-5msUI column (length 30 m, I.D. 25 mm, Agilent). Mass spectra were recorded from 50 to 450 m/z. The injection temperature was 280°C. The oven temperature was increased from 50 to 300°C at 5°C/min, and maintained at 300°C for 5 min.

Metabolites were at first identified by comparison of their retention time with authentic standards. Further identifications were achieved through spectral matching against the National Institute of Standard and Technology (NIST). Matches with a reverse similarity index (RSI) value ≥700 were considered. But some of the matches could not be linked to a clearly defined biological function, and therefore these metabolites were considered unidentified. Individual integrated peak areas of the metabolites were all converted to response ratios relative to the internal standard sucrose. According to four biological replicates, metabolites that changed ≥1.5-fold in relative ratios with a *p*-value < 0.05 (One-way ANOVA) were considered for further analysis. Principal component analysis (PCA) was carried out with MetaGeneAlyse v 1.7.1 (Max Planck Institute of Molecular Plant Physiology, Germany).

## Electronic supplementary material

Additional file 1: Figure S1: Representative PMF and MS/MS spectra of protein spot b3. (PDF 105 KB)

Additional file 2: Figure S2: Additional data for proteins identified by a single peptide. (PDF 119 KB)

Additional file 3: Figure S3: Representative GC-MS total ion chromatograms of metabolites from nondiapause- (N) and diapause-destined (D) larval hemolymph in prediapause phase. (PDF 103 KB)

Additional file 4: Figure S4: Holometabolous development of the cotton bollworm, *H. armigera.* (PDF 1016 KB)
