# Supplementary figures and images for: Proteomic and metabolomic profiles of larval hemolymph associated with diapause in the cotton bollworm, Helicoverpa armigera
Source: BMC Genomics. 2013 Nov 1;14(1):751. doi: 10.1186/1471-2164-14-751 (PMC4046812; doi:10.1186/1471-2164-14-751)

Additional file 1: Figure S1

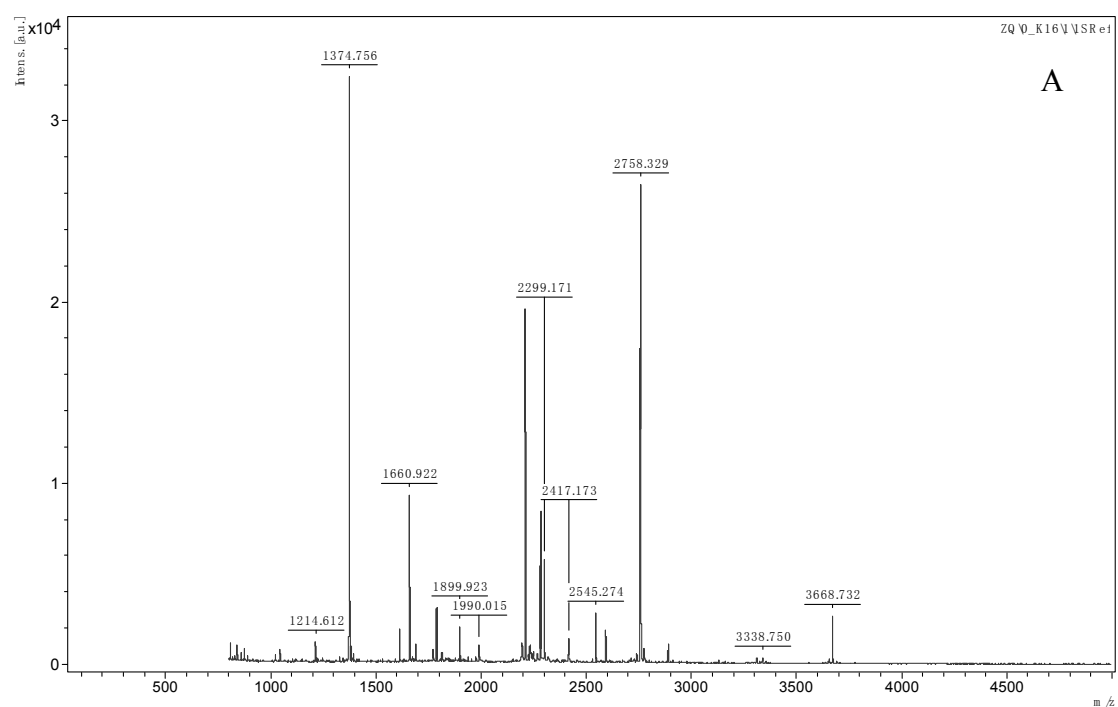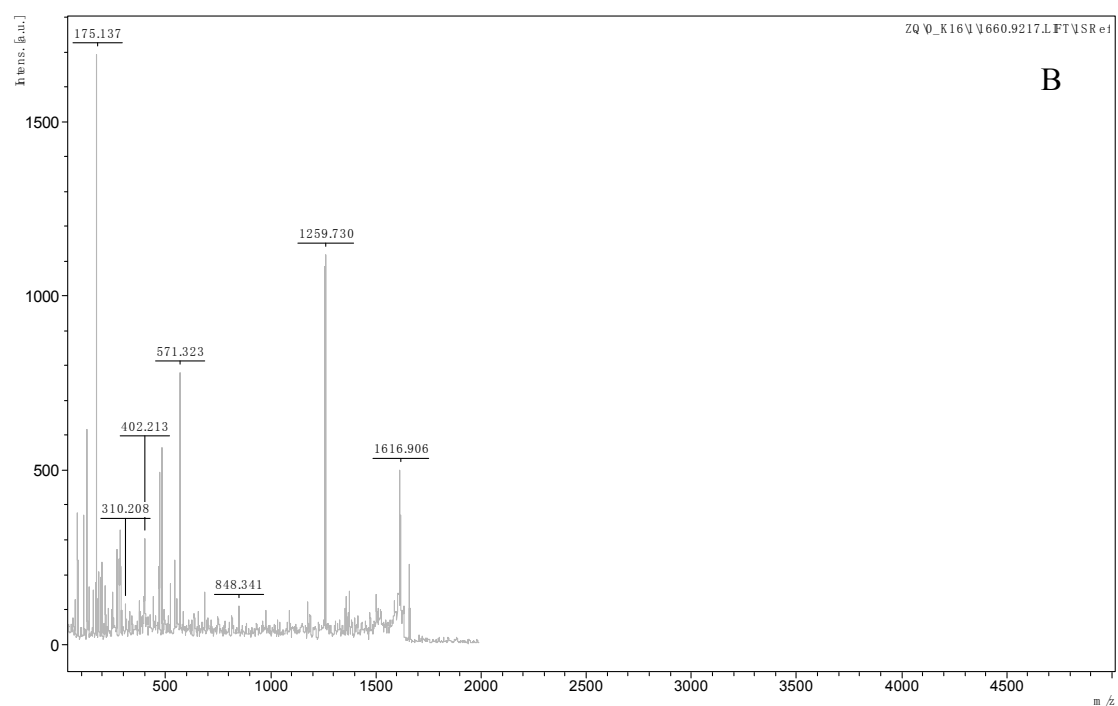

Supplement: Supplementary file 1 — Additional file 1: Figure S1: Representative PMF and MS/MS spectra of protein spot b3. (PDF 105 KB) [file 12864_2013_5472_MOESM1_ESM.pdf]

The induction phase

Mid-late 5th

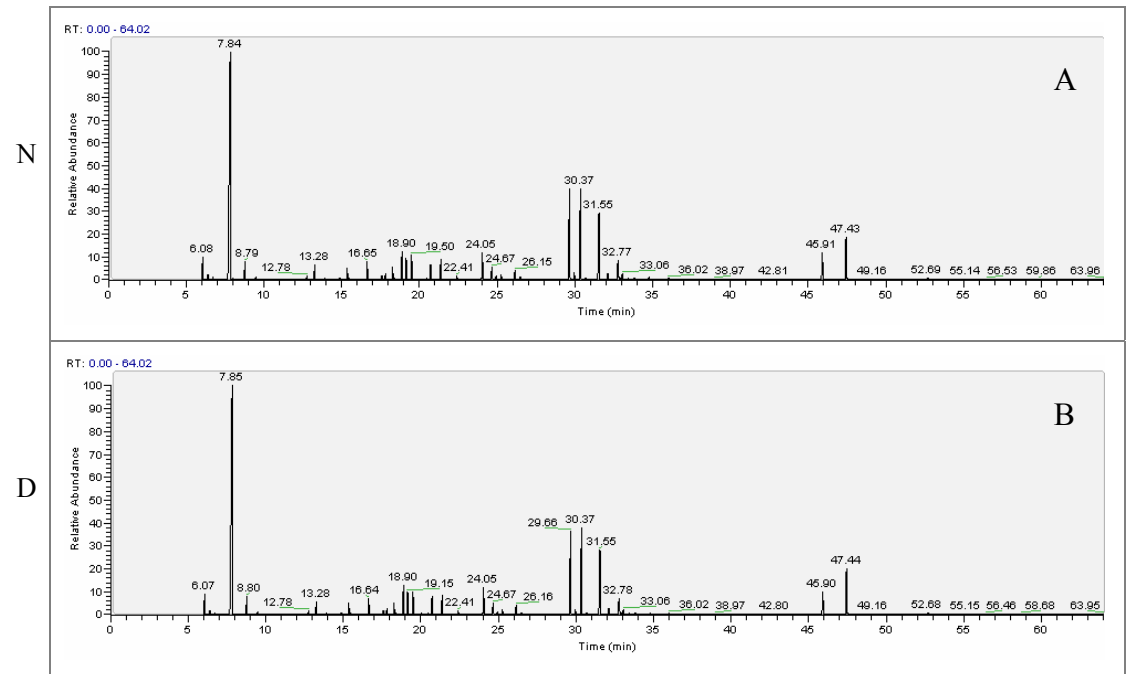

Early 6th

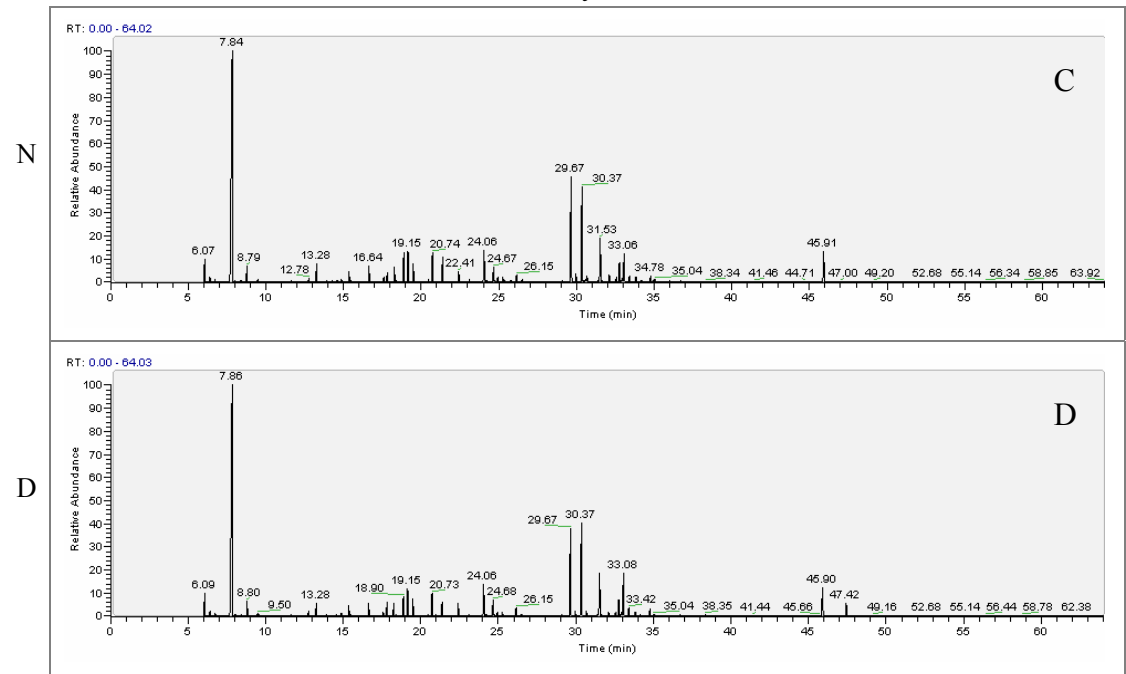

## The preparation phase

### Mid 6th

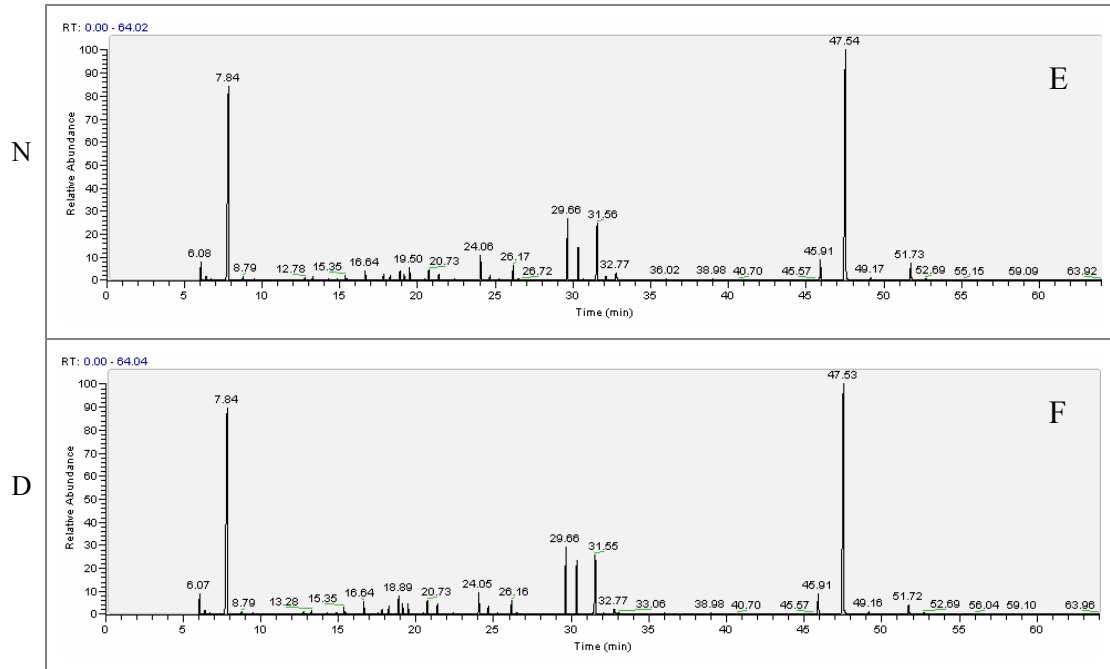

### Late 6th

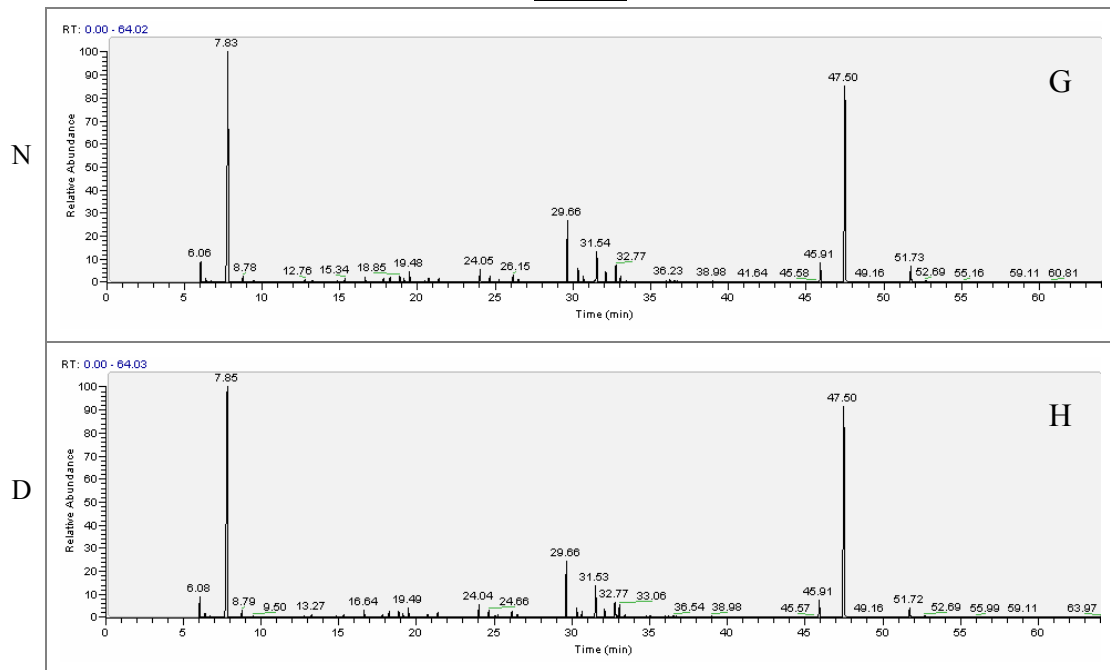

Supplement: Supplementary file 3 — Additional file 3: Figure S3: Representative GC-MS total ion chromatograms of metabolites from nondiapause- (N) and diapause-destined (D) larval hemolymph in prediapause phase. (PDF 103 KB) [file 12864_2013_5472_MOESM3_ESM.pdf]

Additional file 4: Figure S4

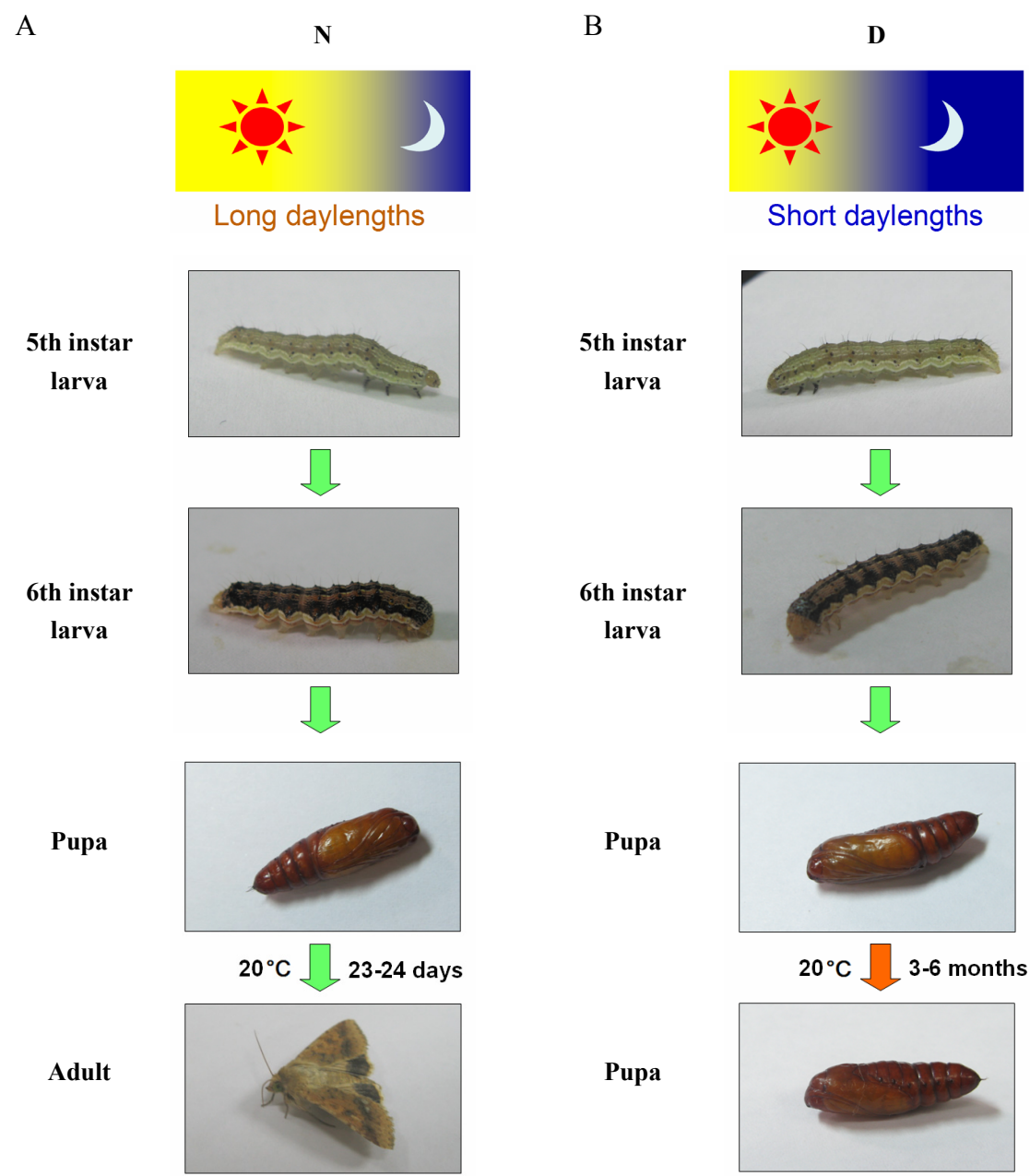

Supplement: Supplementary file 4 — Additional file 4: Figure S4: Holometabolous development of the cotton bollworm, H. armigera. (PDF 1016 KB) [file 12864_2013_5472_MOESM4_ESM.pdf]
