# Supplementary material for: Proteomic and metabolomic profiles of larval hemolymph associated with diapause in the cotton bollworm, Helicoverpa armigera
Source: BMC Genomics. 2013 Nov 1;14(1):751. doi: 10.1186/1471-2164-14-751 (PMC4046812; doi:10.1186/1471-2164-14-751)

Additional file 2: Figure S2

A

Spot no.	Observed	Mr(expt)	Mr(calc)	ppm	Score	Peptide
a2	2210.1688	2209.1616	2209.1280	15.2	79	IPVSLNNLIQHVDYWGEGR

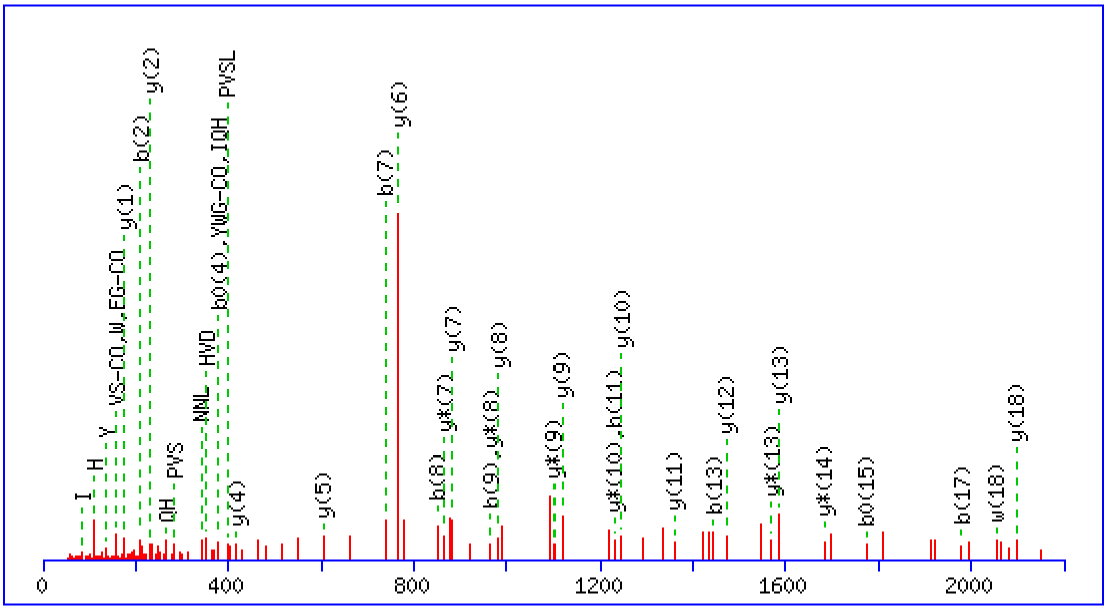

B

Spot no.	Observed	Mr(expt)	Mr(calc)	ppm	Score	Peptide
a3	1656.9591	1655.9519	1655.9246	16.4	105	QPYESDIKVPLVIR

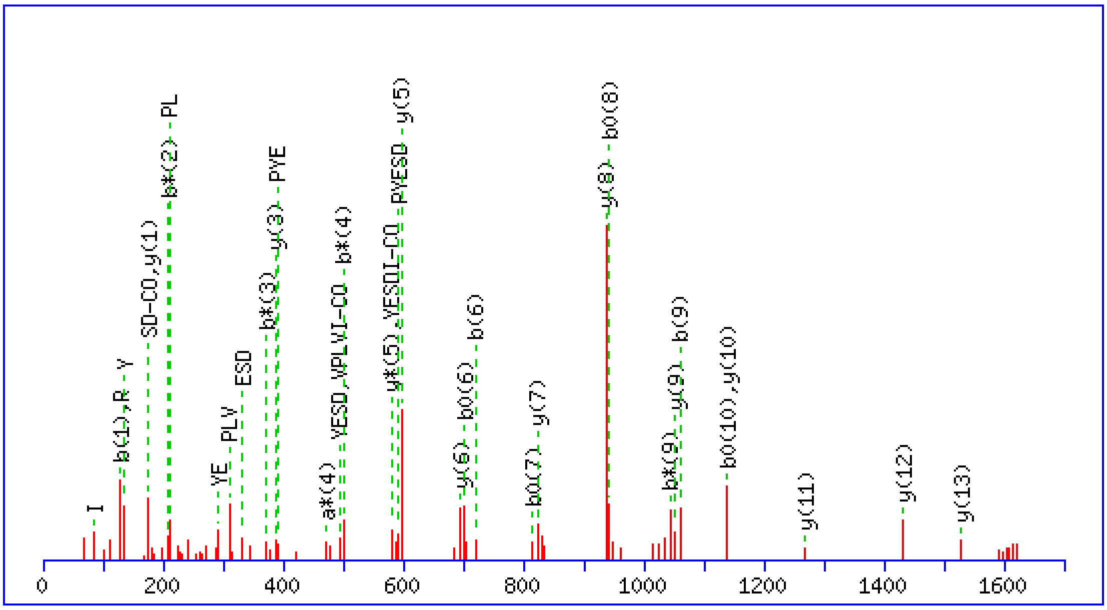

C

Spot no.	Observed	Mr(expt)	Mr(calc)	ppm	Score	Peptide
a4	1656.9352	1655.9279	1655.9246	1.99	114	QPYESDIKVPLVIR

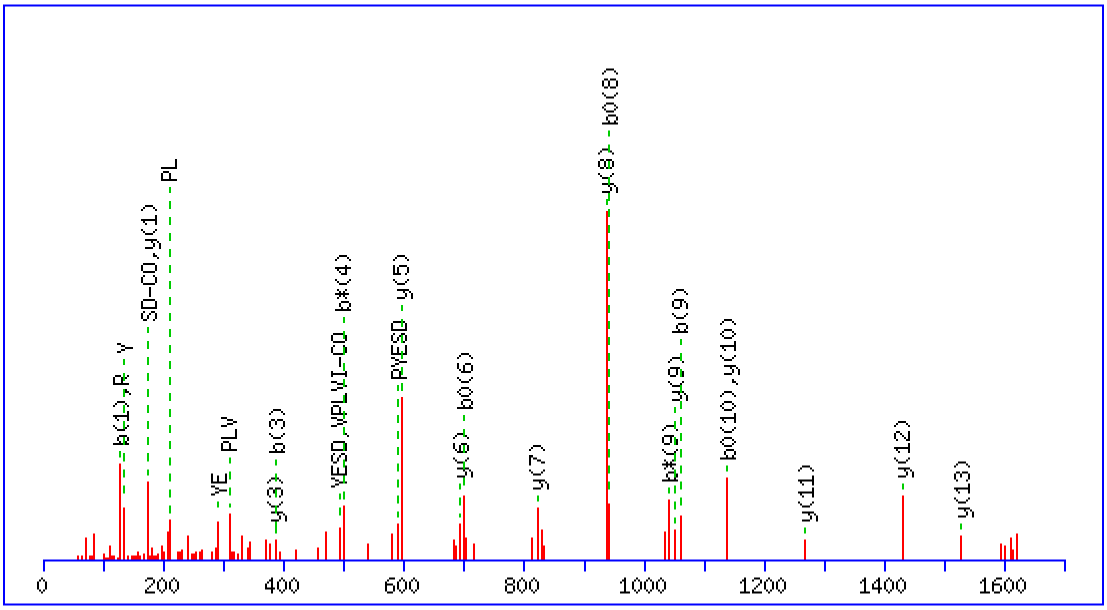

D

Spot no.	Observed	Mr(expt)	Mr(calc)	ppm	Score	Peptide
b5	2478.2043	2477.1971	2477.1679	11.8	49	SQAPEACMGLSGEPLLVSN QFSR

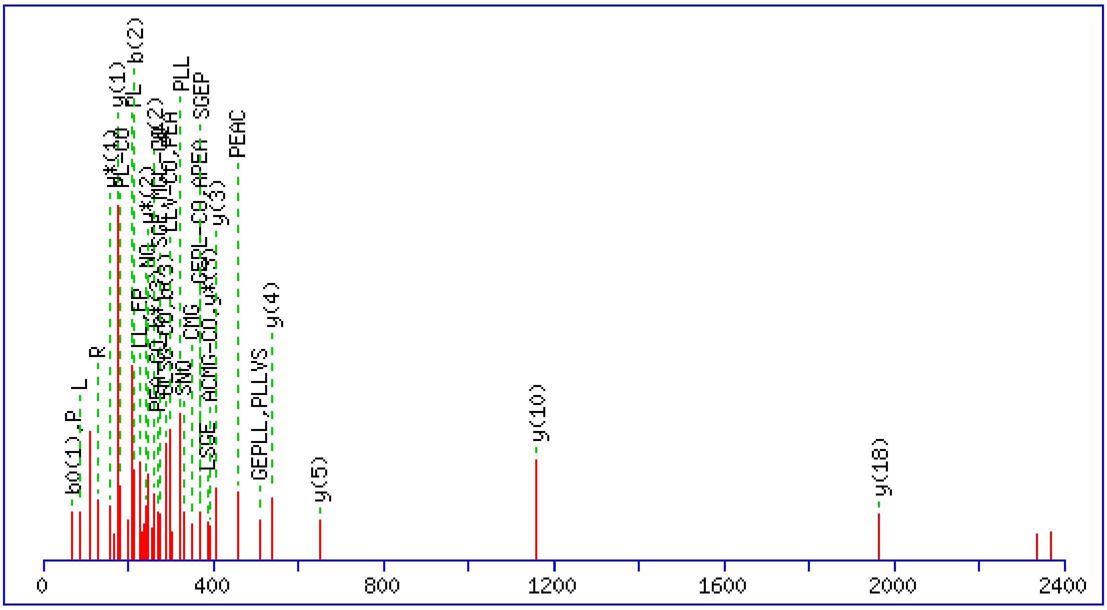

E

Spot no.	Observed	Mr(expt)	Mr(calc)	ppm	Score	Peptide
c3	2114.0283	2113.0210	2113.0228	-0.86	49	IPLVWANDEPDNFGNKER

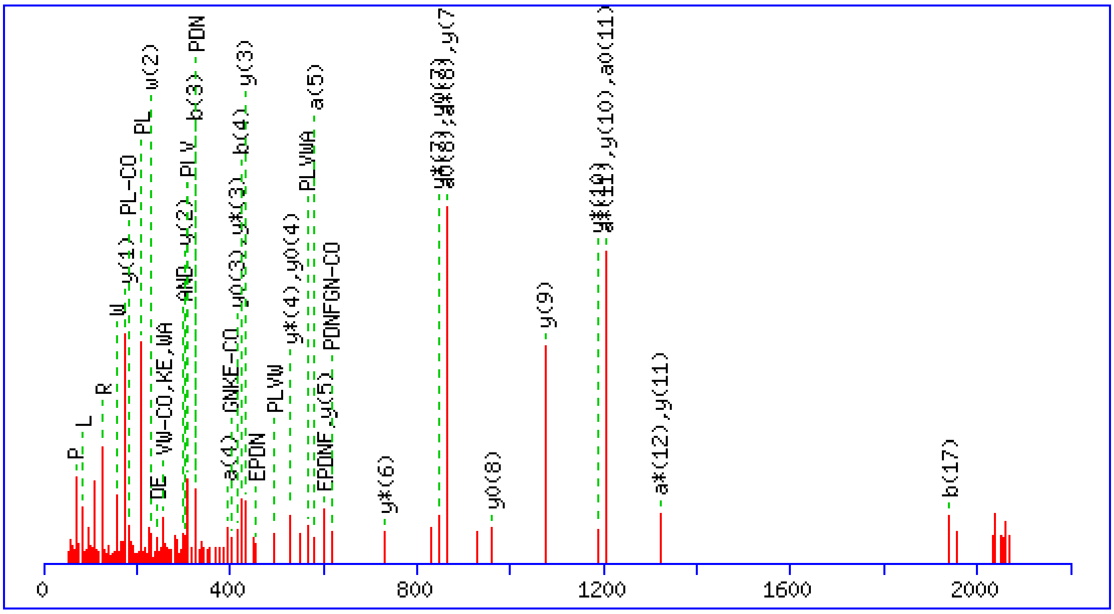

F

Spot no.	Observed	Mr(expt)	Mr(calc)	ppm	Score	Peptide
c9	2326.1768	2325.1695	2325.2117	-18.15	99	NPLLNADAVVTYWLQGGA PAQK

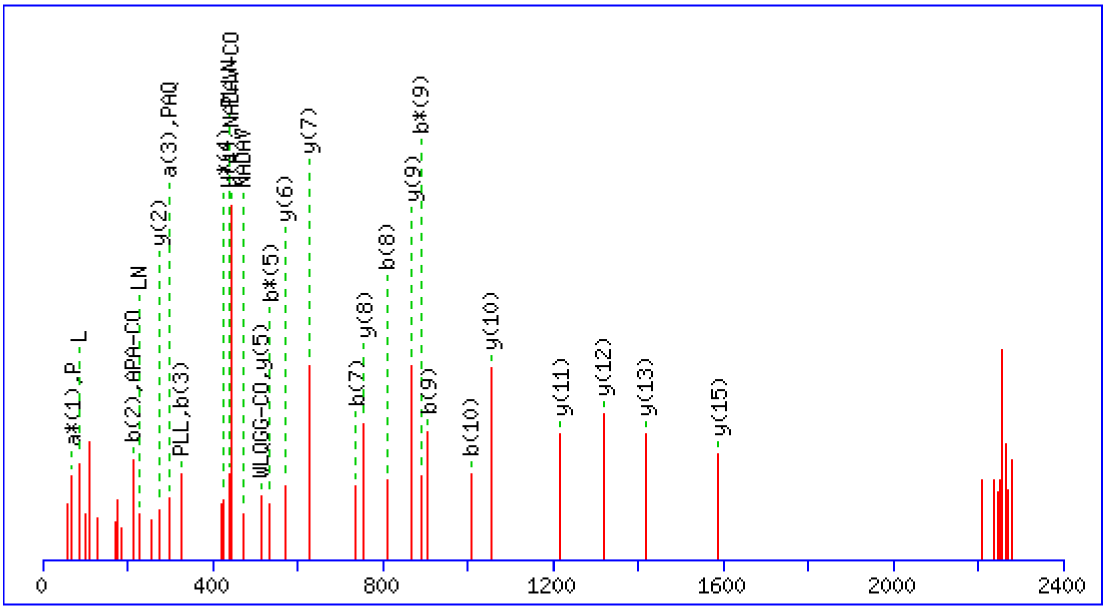

Supplement: Supplementary file 2 — Additional file 2: Figure S2: Additional data for proteins identified by a single peptide. (PDF 119 KB) [file 12864_2013_5472_MOESM2_ESM.pdf]
